# Increasing Metabolic Diversity in Marine Sponges Extracts by Controlling Extraction Parameters

**DOI:** 10.3390/md16100393

**Published:** 2018-10-20

**Authors:** Lina M. Bayona, Melina Videnova, Young Hae Choi

**Affiliations:** 1 Natural Products Laboratory, Institute of Biology, Leiden University, Sylviusweg 72, 2333BE Leiden, The Netherlands; l.m.bayona.maldonado@biology.leidenuniv.nl (L.M.B.); melvidenova@gmail.com (M.V.); 2 College of Pharmacy, Kyung Hee University, Seoul 02447, Korea

**Keywords:** marine organism, extraction, metabolomic diversity, ^1^H NMR, design of experiment

## Abstract

Metabolomics has become an important tool in the search for bioactive compounds from natural sources, with the recent inclusion of marine organisms. Of the several steps performed in metabolomics studies, the extraction process is a crucial step—one which has been overlooked for a long time. In the presented study, a pressurized liquid extraction system was used to investigate the effect of extraction parameters such as pressure, temperature, number of cycles, and solvent polarity on the chemical diversity of the extract obtained from the marine sponge, *Xestospongia*. For this, a full factorial design (2^4^) was performed using a chemical diversity index, which was found to be a suitable tool to determine the efficiency of the extraction process, as the response variable. This index was calculated using a logarithmic transformation of ^1^H NMR signals. Three factors (number of cycles, temperature, and solvent polarity) and two interactions were found to affect the chemical diversity of the obtained extracts significantly. Two individual factors (temperature and solvent polarity) were selected for further study on their influence on sponge metabolites using orthogonal partial least square (OPLS) modeling. Based on the results, the groups of compounds that were most influenced by these parameters were determined, and it was concluded that ethanol as the extraction solvent together with low temperatures were the conditions that provided a higher chemical diversity in the extract.

## 1. Introduction

Drug discovery is an arduous process that requires enormous amounts of time and money and, more importantly, it requires a vast pool of candidates to uncover a lead compound. It has been calculated that between 5000 to 10,000 chemical candidates are needed at the beginning of the drug development process in order to obtain one approved drug [1]. Consequently, the search for a sustainable source which can provide a large number of chemical entities has always been a long-term goal for scientists. Natural products, defined as all the small molecules naturally synthesized by a living organism, have undoubtedly been one of the most prolific sources of bioactive chemicals for drug discovery. For instance, of all the drugs that were approved between 1981 and 2014, over 45% were natural products, natural products derivatives, or synthetic drugs that were inspired by natural products [2].

Information regarding traditional medicine has been collected over centuries from regions such as Sumer, Egypt, and China, and this has led to the use of medicinal plants to treat a wide range of diseases. [3]. Relatively easy access for collection and the wealth of ethnopharmacological information enabled the development of a great number of drugs from plants during the 19th century. Since the beginning of the 20th century there was a surge in the development of modern biotechnology and advancement of scuba techniques, allowing for other natural sources such as microorganisms and marine organisms to present themselves as a rich alternative source of new bioactive compounds with very different metabolic pools to those in plants [4,5,6].

Marine organisms have opened a new era for the discovery of a novel pool of molecules that could be used as drug candidates. Interestingly, many compounds isolated from marine organisms have shown very distinctive chemical characteristics, which could consequently provide a more productive source of chemical entities. For instance, some of these compounds have presented with unique structural features, such as the inclusion of bromine and chlorine atoms by a covalent bond, polyketide compounds, and nonribosomal peptides [7,8]. This makes marine organisms a fascinating source of molecules with a potential to be used as drugs. Nowadays, thousands of new marine natural products with diverse bioactivity are being reported every year. Recently, the FDA approved seven drugs from marine sources for the treatment of different diseases [9]. However, despite the potential and vast level of chemical diversity found in marine organisms, the number of registered new drugs appears to remain low.

The difficulties for the development of new drugs come from the low natural abundance of marine organisms in the wild and restrictions in sample collection. Additionally, marine organisms carry an extremely low concentration of active compounds, consequently requiring large amounts of samples. For example, in the case of sponges, it is estimated that the active compounds correspond to less than 0.4% of the dry weight of the organism [10]. Moreover, the effect of environmental and ecological factors on the production of active compounds is still unclear [11]. To mitigate the adverse effects of these issues, every step during the process of investigating marine organisms should be carried out carefully to minimize the loss of compounds. For these reasons, meticulous attention is required for appropriate sample preparation. Preanalytical processing, including extraction, is a particularly important step in marine organisms. Unlike terrestrial samples, marine samples usually contain more inorganic salts and fatty materials due to their environmental conditions. Additionally, the places from which samples are collected are often remote, which leads to long delays between collection and extraction in the laboratory. Therefore, a robust protocol is required to avoid interferences caused by salt, sample storage, and transportation during the sample preparation.

Following sample collection, extraction is performed in order to dissociate metabolites from the matrix. When extracting natural products, it is not only essential to obtain a high yield of targeted specific metabolites but also a qualitative feature: the number of metabolites to be extracted. Particularly in the case of marine organisms; managing to isolate the large variety of metabolites that are present within the matrix in small quantities is of high importance during the extraction process. This has encouraged increased applications of metabolomics studies to marine organisms as part of the recent trends of life science research. In these holistic approaches, it is important to extract a wide range of compounds to fulfill the aim of acquiring a comprehensive overview of the chemical composition of an organism [12]. Previously, many extraction methods have been suggested for metabolomics study as was reviewed by Mushtaq and colleagues [13]. Additionally, as an alternative to the conventional extraction methods, a comprehensive extraction method using multiple solvents was suggested [14]. However, no methods have been described to target marine organisms specifically.

During extraction, several parameters can influence the efficiency of the process such as solvent polarity, temperature, pressure, the ratio between the samples and solvents, as well as mechanical assistance. The most suitable choice of solvent is crucial for effective extraction, and the selection of the wrong extraction conditions could have a dramatic effect on the extraction yield, e.g., the yield of the extraction of sulfur compounds could be diminished by 90% depending on the chosen solvent [15]. The relationship of other factors such as temperature and pressure with the extraction efficiency have been previously ignored, despite their importance. Although it has been mainly used in terrestrial plants, pressurized extraction can increase the efficiency of extraction. This system combines the use of liquid solvents at elevated temperature and pressure, which could allow to reduce extraction time and maximize the contact between sample and solvent and consequently results in improving the extraction efficiency [16,17]. Furthermore, protocols for the investigation of marine natural products require some preanalytical steps in order to ensure the reproducibility of the results. These steps include desalting of the crude extracts using solid phase extraction (SPE) cartridges as well as the removal of lipid contents using liquid–liquid partition [18,19,20].

In this study, several extraction parameters were investigated on marine organisms, particularly the effect on the metabolic diversity of the extract. A marine sponge was selected as a model organism, as sponges (Porifera) are known to have the highest number of newly reported compounds amongst marine organisms, illustrating the tremendous chemical diversity that this phylum contains [5,6]. Amongst the sponges, *Xestospongia* is an excellent example from this phylum, as over 350 different compounds have been reported in this sponge, including alkaloids, terpenoids, quinones, steroids, and brominates fatty acids [21]. Additionally, the wide distribution of Xestospongia in tropical oceans around the world, including the Indo-Pacific and Atlantic oceans, makes it an exciting organism for further studies of its chemical diversity [22].

In this research, several parameters were examined, including the temperature, pressure, cycles number, and solvent polarity using a pressurized extraction system. A design of experiments (DOE) of a full factorial design was used, and the diversity of chemicals measured was selected as the response variable. For the measurement of chemical diversity, proton nuclear magnetic resonance (^1^H NMR) was applied to the extracts and the diversity index was calculated. Based on the results, the most influential parameters and the interaction between the tested parameters were established. For the next approach to investigate the specific effect on the individual groups of metabolites, two of the most influential parameters were selected, and the chemical data obtained by ^1^H NMR were correlated with the selected factors by a multivariate data analysis; orthogonal partial least square (OPLS) modeling.

## 2. Results and Discussion

To study the correlation between the extraction parameters and metabolic diversity, a pressurized extraction system was employed by controlling the following parameters pressure, temperature, solvent polarity, and cycle number. Of the large number of parameters involved in the extraction process, these four parameters were chosen to evaluate their effect on the chemical diversity of the extract. To identify the most influential factor and the interactions between factors on the resulted chemical diversity, a full factorial design (2^4^) was applied to the chemical data.

Solvent polarity was selected as a parameter as it is usually the most influential factor on the yield of the extraction. From the numerous available solvents that are used for the extraction of marine organisms, e.g., *n*-hexane, dichloromethane, ethyl acetate, methanol, ethanol, isopropanol and other mixtures of solvents, ethanol, and dichloromethane were selected, and their ratio was used as variables in DOE [23]. This allows for an examination of two of the most used solvents in marine organism extraction.

Extraction temperature is well-known for increasing the rate of the diffusion of metabolites from the matrix into the solvent. Here, the low and high levels were set to 30 °C and 70 °C, respectively. The low temperature was chosen as it is close to room temperature and many extractions are performed at this temperature. The high temperature was chosen as it is close to the boiling point of ethanol at atmospheric pressure. Additionally, the speed extraction system uses nitrogen as a gas to increase the pressure. For this reason, the temperature can be increased without the risk of degradation of the sample by oxidation. With regards to the pressure parameter, the use of high pressures during extraction is expected to reduce the extraction time as high pressure forces the solvent into the matrix [24]. In this study, the pressure was set at 50 and 100 bars for the low and high levels, respectively. These values were selected based on the technical characteristics of the machine; the lowest working pressure for the extraction system is 50 bar. Furthermore, the high level was set to 100 bars as some reports state the successful use of a pressurized extraction system at this pressure level [23,24].

As the last parameter, the cycles number for the extraction was evaluated. This parameter is a measurement of the ratio between the solvent and sample that should be used to perform the extraction. The metabolites that have a higher affinity for the solvent can saturate the solvent, which prohibits the extraction of other compounds and potentially lowers the diversity of metabolites that can be extracted. In this case, the number of cycles was set to one or three cycles for the low and high settings, respectively. After each extraction cycle, fresh solvent was added to the sample.

### 2.1. Chemical Diversity Index

One of the most vital necessities of natural products, including marine organisms, is providing a wide range of chemicals. Particularly in drug discovery, diversity is the critical issue in a chemical pool. To explore the chemical diversity of natural products, chemical profiling tools like metabolomics are being actively applied to many marine organisms. The quality of metabolomics results is dependent on extraction step. To produce a broad range of metabolites, the extraction process must cover as many metabolites as possible in the target organisms.

To quantify the diverse array of extracted chemicals, an index was developed in this study based on the ^1^H NMR analysis of the samples. ^1^H NMR can detect various groups of metabolites and all of the signals are highly proportional to the molar concentration of the compounds, which could make it possible to quantitatively compare the signals within the same sample. Therefore, the change in the extraction efficiency of the diverse groups of metabolites in different extraction conditions can be clearly viewed [25].

**Figure 1:**
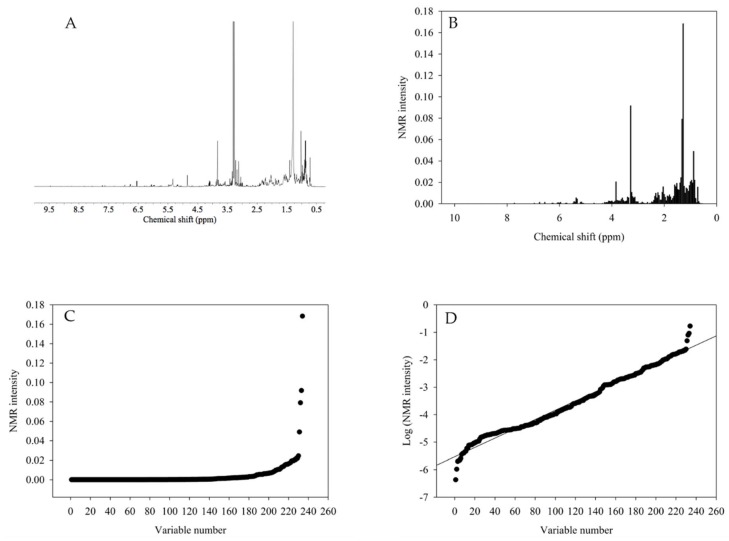
Schematic of the data processing for the calculation of the chemical diversity index. (**A**) NMR-processed spectra, (**B**) NMR spectra after bucketing, (**C**) plot of the NMR intensity against the variable number, and (**D**) the plot of the logarithm of the ^1^H NMR intensity against the variable number.

For the calculation of this index (as exemplified in Figure 1), firstly, the ^1^H NMR spectra (Figure 1A) was integrated or bucketed in a certain range, e.g., 0.04 ppm (Figure 1B) and the integrated signals were sorted from the highest to the lowest (Figure 1C). Some signals, however, were shown to have an extremely high intensity which resulted in an exponential curve (Figure 1C). This happened because a few signals, which mainly corresponded to fatty acids, were very high in comparison to others, such as the aromatic region. To solve the problems of the signals, the ^1^H NMR data were transformed logarithmically (Figure 1D) and the transformed signal intensities fit the 1st order line. The inverse of the 1st order fitting curve was referred to as the chemical diversity index. A higher value of the slope indicates that a specific group of metabolites was extracted preferentially, whilst a slope close to zero indicates that all of the metabolites are extracted with the same efficiency. For further evaluation of the extraction factors, the calculated chemical diversity index was used as the response variable in the DOE analysis.

### 2.2. Evaluation of Extraction Parameters Using a Design of Experiments

To investigate the effects of solvent polarity, temperature, pressure, and the number of cycles (factors) on the resulted chemical diversity (response), a pure screening DOE was applied to the data set. The response (chemical diversity index) was modeled using a constant (β_0_), four coefficients of individual parameters (β_0_–β_4_), and six coefficients of interaction parameters (β_12_, β_13_, β_14_, β _23_, β_24_, and β_34_). As shown in Table 1 (the significant values are marked with *), three factors and two interactions between the factors were found to significantly affect the extraction process. Firstly, the number of cycles factor displayed a positive correlation with the extraction process, indicating that more cycles allow for the extraction of more diverse metabolites in the extract. This might be due to the fact that metabolites with low solubility could gradually be extracted by the newly added solvent during each cycle.

The second factor of interest was the polarity of the solvent in the system of ethanol and dichloromethane. An increase in the solvent polarity was found to correlate positively with the diversity of the extracted compounds. It shows that ethanol may be a more suitable solvent for the extraction of a wide range of compounds than mixtures of ethanol and dichloromethane. Although a large percentage of sponge metabolites are lipophilic fatty chemicals, ethanol may be more efficient to extract polar secondary metabolites, which resulted in higher number of extracted metabolites. The final factors that were selected were temperature and pressure. Interestingly, a higher temperature of the extraction procedure resulted in a decrease of the chemical diversity of the extract. On the other hand, in the case of pressure, it was not found to be significantly related to the extracted chemical diversity of the extraction. In general, the physical properties of liquids are not affected by pressure as much as gas, including solvent power.

The interaction between the number of cycles and the temperature in the extraction process also has a significant effect on the outcome of chemical diversity. At low temperature, the extract displayed almost no difference between the chemical diversity depending on the number of cycles. However, at higher temperatures, the diversity decreased dramatically if one cycle was used for extraction. This may be related to the penetration efficiency of the solvent through the matrix. At higher temperatures, lipids from the matrix are extracted more efficiently when compared to an extraction at lower temperatures. Due to this effect, other metabolites are not extracted, and the chemical diversity is reduced. Therefore, at higher temperatures more cycles are required to achieve a similar chemical diversity in the extraction.

A similar effect was found in the interaction between the number of cycles and the pressure of the extraction. At lower pressures, the chemical diversity of the extract was smaller if one cycle was used for the extraction. However, at higher pressures one cycle displayed a higher chemical diversity whilst three cycles showed a decrease in the diversity.

For the next steps, solvent polarity and temperature were selected for further investigation with regards to which groups of metabolites were more influenced by these selected parameters.

### 2.3. Effect of the Solvent Polarity and Temperature on an Individual Group of Metabolites

To investigate the effect of solvent polarity and temperature in detail, a supervised multivariate data analysis (MVDA), OPLS modeling, was applied to the ^1^H NMR data using the percentage of ethanol and temperature as Y-variables.

**Figure 2:**
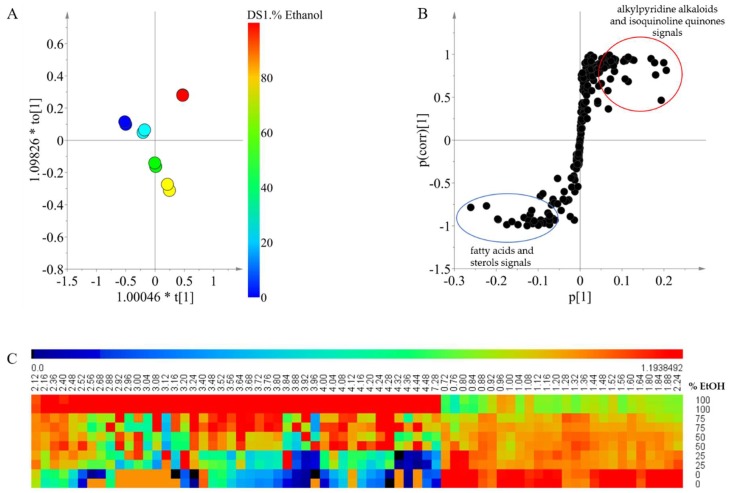
Orthogonal partial least square modeling using ^1^H NMR data of Xestospongia extracts combined with solvent polarity (the percentage of ethanol) (**A**: score plot, **B**: S-plot) and heat map of the relative intensities of the discriminant signal (**C**). The extractions were performed at 30 °C. Red circle: Metabolites with higher polarity. Blue circle: metabolites with lower polarity.

Figure 2 shows the effect of the polarity of the extraction solvent on the resulting metabolites, for which the solvents with different ethanol percentages were used at the same temperature (30 °C). In the score plot, clear differences between the samples with different percentages of ethanol were found (Figure 2A), and the OPLS model was validated as *Q*^2^ was 0.984 and the cross- validation analysis of variance (CV-ANOVA) test had a *p*-value < 0.05. The results show that there is a significant change in the chemical composition of the extract, depending on the solvent polarity that is employed.

To analyze the correlation between the signals and the separation according to the solvent composition observed in the OPLS modeling, an S-plot was made (Figure 2B). For the extracts that were prepared using higher ratios of dichloromethane, the signals between δ 0.72 and δ 2.24 displayed a higher intensity. Signals in this range correspond to fatty acids and sterols which have been widely reported in *Xestospongia* [26,27,28]. These compounds are very nonpolar and are therefore expected to be extracted more efficiently by dichloromethane when compared with ethanol. In the case of a higher percentage of ethanol for the extract, the intensity of signals between δ 2.5 and δ 4.5 was higher than other signals. Many of the signals within the range may be related to functional groups such as methyl groups bound to oxygen or nitrogen atoms present in alkylpyridine alkaloids and isoquinoline quinones, as previously reported in *Xestospongia* [29,30].

The overview of metabolic profile obtained by the solvents at different polarities is shown in the form of a heat map (Figure 2C). In this map, signals of a higher chemical shift which were related to an extraction solvent of high polarity displayed intensities that were very close to zero in a low polarity extraction solvent. This indicates that the compounds with high polarity were not extracted at all using solvent mixtures with a high content of dichloromethane. Moreover, signals at lower chemical shifts related to a lower polarity of the extraction solvent are also extracted using solvent mixtures with a higher content of ethanol, but they are extracted less efficiently when compared to the dichloromethane extracts. This explains why the increase in the solvent polarity had a positive effect on the chemical diversity of the extract. Although ethanol has higher affinity for polar compounds, it is also able to extract compounds with very low polarity from the matrix.

### 2.4. Effect of the Temperature

**Figure 3:**
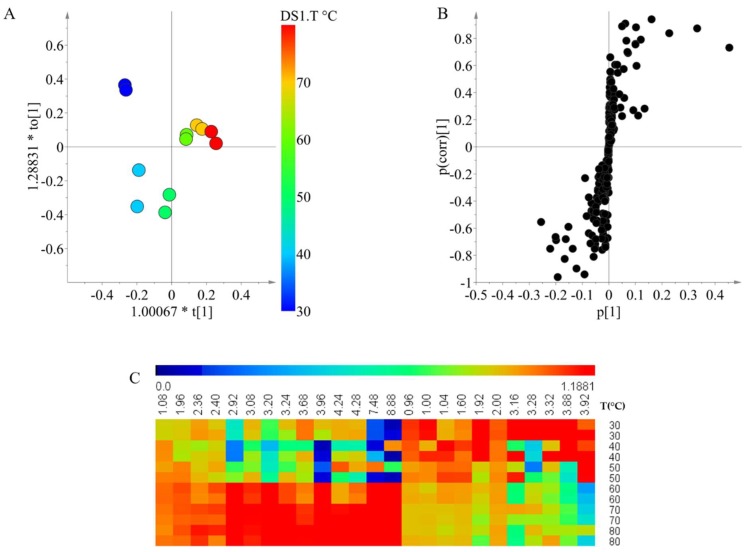
Orthogonal partial least square modeling using ^1^H NMR data of Xestospongia ethanol extracts varying the temperature (**A**: score plot, **B**: S-plot) and heat map of the relative intensities of the discriminant signal (**C**).

The temperature was the second selected factor for the detailed study on the relationship with the individual groups of metabolites. For the temperature effects, 100% ethanol was used as the extracting solvent by varying temperatures between 30 and 80 °C in 10 °C steps. The score plot of the OPLS modeling showed that the metabolites are greatly influenced by temperature; the chemical composition of extractions between 30 and 50 °C were grouped together as the chemical composition was similar, whilst the extraction between 60 and 80 °C were grouped into a second group (Figure 3A). The model was validated, as *Q*^2^ was 0.953 and the CV-ANOVA test had a *p*-value < 0.05.

To identify the signals that were correlated within each group of temperatures, an S-plot was constructed (Figure 3B). For the low temperatures, signals between 0.96 and 3.92 ppm were distinguished as significant. Similarly, in the case of extractions at high temperature, signals in the same region as well as few signals in the aromatic region were correlated. This indicates that the differences in the extraction caused by the temperature do not correspond to a family of compounds as the region of the significant signals is almost the same. Instead, the difference is caused by specific compounds that are extracted differently depending on the temperature.

Lastly, to compare the relative intensities of the discriminant signals, a heat map was produced. Here, it is possible to observe the clear difference between the low temperature (below 50 °C) and high temperature (above 60 °C) extractions. Unlike the solvents, a preferential extraction is not clearly observed. This selectivity of extraction in the compounds depending on the temperature could be related to the increase of the chemical diversity at low temperatures as showed in the first experiment. At low temperatures, all of the compounds are extracted with similar efficiency whilst at higher temperatures, a specific group is preferred. In that sense, for analysis that requires a holistic overview of the chemical composition of the samples, lower temperatures for extraction should be preferred.

## 3. Materials and Methods

### 3.1. Sponge Collection

Sponges *Xestospongia* sp. were collected from the inner coral reef in Martinique, in September 2016, and the samples were preserved in ethanol for the transportation, the samples were storage at −20 °C. The specimens were identified by Nicole de Voogd (National Museum of Natural History, The Netherlands). The ethanol used for storage was removed by filtration. The samples were frozen under liquid nitrogen and freeze-dried using a Labconco FreeZone 4.5 plus freeze dryer. The dried samples were ground and pooled into a single sample batch (17.9 g dried weight). The same samples were used for all the experiments in the study.

### 3.2. Experimental Design

In this study, a full factorial design with n = 4 was used to identify if the pressure, temperature, number of cycles, and solvent polarity influence the chemical diversity of the extract. Two levels were defined for each variable, − and +. The temperature was changed between 30 and 70 °C and the pressure was changed between 50 bar and 100 bar. Cycles number was changed between 1 and 3. The solvent was changed between dichloromethane/ethanol 1:1 (*v*/*v*) and 100% ethanol. The detailed conditions of the experiments are shown in Table 2. The experiments were carried out in triplicate.

For the second part of experiments conditions of temperature and solvent polarity varied after pressure was set at 90 bar and the number of cycles was three. For the temperature evaluation, 100% ethanol was used with temperature variation of 30, 40, 50, 60, 70, and 80 °C. For the solvent polarity evaluation, the temperature was set at 30 °C and the solvent proportion of dichloromethane/ethanol was changed between 100% dichloromethane, 75:25 (*v*/*v*), 1:1 (*v*/*v*), 25:75 (*v*/*v*), and 100% ethanol. All of the experiments were carried out by duplicate.

### 3.3. Pressurized Solvent Extraction

Pressurized solvent extraction was performed using a Speed Extractor E-916 (BÜCHI Labortechnik AG, Flawil, Switzerland). Six stainless steel extraction cells (10 mL) were used at a time, placing two circular glass fiber filters on both ends and a metal frit at the bottom. The cells were packed with 9 g of Quartz sand (diameter: 0.3–0.9 mm, BÜCHI Flawil, Switzerland), mixed with the ground samples (150 mg and 200 mg for 1st and 2nd experiments, respectively).

### 3.4. ^1^H NMR Analysis

From the extract obtained from the Speed Extractor system, 1.5 mL was taken to dryness. The residue was resolved in CH_3_OH-*d*_4_ with hexamethyldisiloxane (HMDSO) as the internal standard. The ^1^H NMR spectra were measured at 25 °C in an AV-600 MHz NMR spectrometer (Bruker, Karlsruhe, Germany), operating at the ^1^H NMR frequency of 600.13 MHz, and equipped with a TCI cryoprobe and Z gradient system. For internal locking, CH_3_OH-*d*_4_ was used. A presaturation sequence was used to suppress the residual water signal, using low power selective irradiation at the H_2_O frequency during the recycle delay.

### 3.5. Data Prepossessing and Statistical Analysis

The resulting spectra were phased, baseline corrected, and calibrated to HMDSO at 0.06 ppm using TOPSPIN V. 3.0 (Bruker Karlsruhe, Germany). The NMR spectra were bucketed using AMIX 3.9.12 (Bruker BioSpin GmbH, Rheinstetten, Germany). Bucket data was obtained by spectra integration at every 0.04 ppm interval from 0.20 to 10.02 ppm. The peak intensity of individual peaks was scaled to the total intensity of the buckets. The regions between 3.32 to 3.28, 4.9 to 4.8, 3.62 to 3.57, and 1.15 to 1.19 ppm were excluded from the analysis because they correspond to solvent residual signals.

The chemical diversity index was calculated using the buckets from the ^1^H NMR spectra. The buckets were organized from high to low, and the logarithm of the bucket was plotted against the order number. These plots are straight lines and the inverse of the slope of these lines corresponds to the chemical diversity index that was used as the response variable in the full factorial design. The statistical analysis was performed using MODDE software (v 12.0.1, Sartorius stedim, Goettingen, Germany).

To analyze the effect of changing the value of one separate factor at the time (temperature and solvent) the buckets that were obtained from the NMR spectra were organized in a matrix and multivariate data analysis was performed using SIMCA-P software (v.15.0.2, Sartorius stedim, Goettingen, Germany). Principal component analysis PCA and orthogonal projections to latent structures OPLS were performed.

## 4. Conclusions

The extraction process is a crucial step for further detection of metabolites in any natural products study, particularly for metabolomics approaches. The chemical diversity index is a novel tool to quantify the chemical diversity of crude extracts and was successfully used to study the effects of different parameters during the extraction process. From the parameters studied, the number of cycles, solvent polarity, and temperature affected the chemical diversity of the obtained extract significantly. Additionally, the interaction of temperature with the number of cycles and number of cycles with pressure also have a positive and negative effect on the extract, respectively. Lastly, with the second group of experiments, it was possible to confirm that 100% ethanol together with low temperatures were the best conditions to perform the extraction.

## Figures and Tables

**Table 1:** Analysis of the coefficients values of the model using the chemical diversity index as the response variable.

Model Parameter	Regression Coefficients	Coefficient Values
Constant	β_0_	2.68 × 10^1^
Temperature	β_1_	−2.83 × 10^−1^ *
Pressure	β_2_	−2.79 × 10^−4^
Cycles	β_3_	5.06 × 10^−1^ *
Solvent polarity	β_4_	3.27 × 10^−1^ *
Temperature-Pressure	β_12_	1.91 × 10^−1^
Temperature-Cycles	β_13_	3.60 × 10^−1^ *
Temperature-Solvent polarity	β_14_	1.11 × 10^−1^
Pressure-Cycles	β_23_	−2.28 × 10^−1^ *
Pressure-Solvent polarity	β_24_	−1.94 × 10^−2^
Cycles-Solvent polarity	β_34_	−2.92 × 10^−2^
R^2^		6.70 × 10^-1^
R^2^ adjusted		5.81 × 10^-1^
*p* value		2.20 × 10^−6^
Lack of fit		5.72 × 10^−2^

* significant at *p* < 0.05.

**Table 2:** Experimental design for the extraction of Xestospongia using pressurized extraction system.

Number of Experiments	X1 (Temperature)	X2 (Pressure)	X3 (Number of Cycles)	X4 (Solvent)
1	−	−	−	−
2	+	−	−	−
3	−	+	−	−
4	+	+	−	−
5	−	−	+	−
6	+	−	+	−
7	−	+	+	−
8	+	+	+	−
9	−	−	−	+
10	+	−	−	+
11	−	+	−	+
12	+	+	−	+
13	−	−	+	+
14	+	−	+	+
15	−	+	+	+
16	+	+	+	+
